# IRF8 configures enhancer landscape in postnatal microglia and directs microglia specific transcriptional programs

**DOI:** 10.1101/2023.06.25.546453

**Published:** 2023-08-17

**Authors:** Keita Saeki, Richard Pan, Eunju Lee, Daisuke Kurotaki, Keiko Ozato

**Affiliations:** 1: Division of Developmental Biology, National Institute of Child Health and Human Development, National Institutes of Health, Bethesda, MD, 20892; 2: MD-PhD Candidate in Neurobiology and Behavior, Columbia University, School of Medicine, New York, NY; 3: International Research Center for Medical Sciences, Kumamoto University, 860-011 Kumamoto City, Japan

**Keywords:** IRF8, microglia maturation, enhancer, Alzheimer’s disease

## Abstract

Microglia are innate immune cells in the brain. Transcription factor IRF8 is highly expressed in microglia. However, its role in postnatal microglia development and the mechanism of action is unknown. We demonstrate here that IRF8 binds to enhancer regions of postnatal microglia in a stepwise fashion reaching a maximum after day 14, which coincided with the initiation of microglia function. Constitutive Irf8 deletion led to the loss of microglia identity gene expression and aberrant induction of Alzheimer’s disease and neurodegeneration associated genes. Conditional Irf8 deletion in adult microglia showed similar transcriptome profiles, revealing the requirement of continuous IRF8 expression. Additional genome-wide analyses showed IRF8 is critical for setting microglia-specific chromatin accessibility and DNA methylation patterns. Lastly, in the 5xFAD mouse AD model, Irf8 deletion lessened the formation and spread of amyloidβ plaques, thereby reducing neuronal loss. Together, IRF8 sets the microglia epigenome landscape, required for eliciting microglia identity and function.

## Introduction

Microglia elicit innate immunity in the brain, providing antimicrobial protection^1, 2^. Microglia also promote brain development through synaptic pruning, supporting neuronal survival, and vascular formation^3, 4, 5, 6^. Microglia continuously surveil the entire brain and respond to changes in their environment^7, 8, 9^. Dysregulation of microglia is associated with neurodegenerative diseases such as Alzheimer’s disease (AD)^10, 11^.

Microglia arise from the yolk sac-derived progenitors and expand in SPI1 (PU.1) and IRF8-dependent manner^12, 13^. Transcription factors, such as Sall1, Smad3, and Mafb, subsequently contribute to microglial maturation^14, 15, 16^. Importantly, microglia do not elicit full function in early postnatal stages and become mature after day 14 (P14)^17^.

IRF8 is a DNA binding transcription factor that binds to ISRE, and composite ETS/ISRE, the latter by interacting with PU.1^18^. IRF8 is expressed in developing myeloid cells in bone marrow, Ly6C+ macrophages, and CD8+ or plasmacytoid dendritic cells in the periphery^19, 20^. IRF8 is essential for innate resistance against various pathogens^21^. Constitutive Irf8 knockout (IRF8KO) mice are susceptible to infection while having excess neutrophils^22^. In the brain, IRF8 is expressed only in microglia, except for a few macrophages^23^. Besides participating in early microglia development, IRF8 takes part in responding to cytokines and pain injury^13, 24, 25^. However, the mechanism of postnatal microglia maturation is largely unknown^14, 17^.

We show that IRF8 plays a decisive role in this process. CUT&RUN assay revealed that IRF8 binds to intergenic and intronic regions of postnatal microglia gradually reaching a maximum after P14, which led to the formation of microglia specific enhancers. IRF8 occupancy was a prerequisite for setting chromatin accessible sites and DNA methylation patterns unique to microglia. Establishment of chromatin landscape coincided with the acquisition of microglia identity and function, as evidenced by transcriptome analyses of constitutive and conditional IRF8KO cells. Deletion of IRF8 caused unexpected induction of Alzheimer’s disease associated genes and altered pathogenesis of AD in a mouse model. Together, IRF8 defines epigenetic landscape in postnatal microglia thereby directing their transcriptome profiles.

## Results

### IRF8 binds to the microglia genome during postnatal development in a stepwise fashion

The IRF8’s role in postnatal development and adult microglia remains unclear. To gain insight into this question, we sought to identify genome wide IRF8 binding sites in postnatally developing microglia. To first verify IRF8 expression in postnatal microglia, we examined Irf8-GFP knock-in mice that express endogenous IRF8 fused to GFP^26^. FACS analyses of microglia from P9, P14, and P60 (adult) showed that IRF8 is expressed at similar levels, higher than that in peritoneal macrophages (Fig. S1A, S1B).

**Figure 1:**
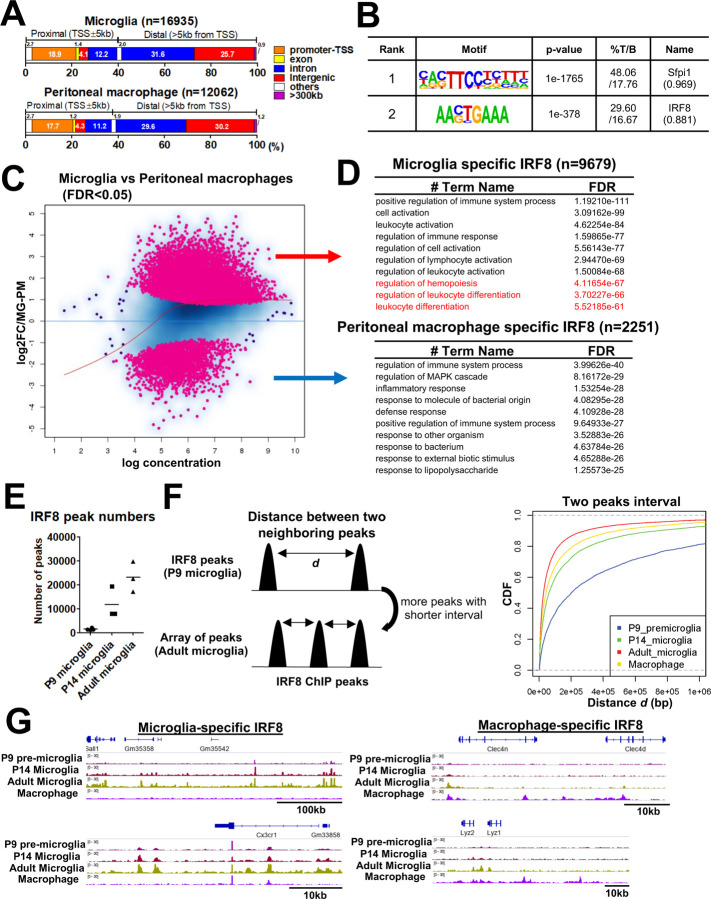
Microglia specific IRF8 occupancy increases postnatally A. Genome wide distribution of IRF8 peaks in adult microglia (top) and peritoneal macrophages (bottom). The genomic position of each peak was determined with HOMER. B. HOMER *de novo* motif analysis for IRF8 binding sites in microglia. Motifs with a match score >0.85 are shown. A motif “Sfpi” is equivalent to PU.1/IRF motif. C. An MA plot visualizing the difference between microglia IRF8 peak intensity and that of peritoneal macrophages (n=3). The differential peaks with FDR<0.05 are indicated in pink. D. Top 10 Gene ontology terms for microglia specific IRF8 peaks (top) and macrophage specific IRF8 peaks (bottom), analyzed with GREAT. Sequences covering 500kb distal from the TSS to 1kb from TES were set as an associating region. The terms of the GO biological process and the corresponding binomial FDR values were shown. E. The number of IRF8 peaks identified in P9, P14, and adult microglia. F. Schematic illustration of the distance between two neighboring IRF8 peaks; the shortening peak intervals represent an array-like appearance of peaks (left). The CDF plot shows that the frequency of short interval peaks increases along with the postnatal microglia development and more than peritoneal macrophages. (right; p=2.2×10^−16^, Kolmogorov-Smirnov two-sided Test). G. IGV examples of IRF8 peaks in developing and adult microglia and peritoneal macrophages at microglia specific genes (Sall1 and Cx3cr1; left) or macrophage specific genes (Clec4n, Lyz1, and Lyz2; right).

CUT&RUN experiments were carried out for microglia isolated from Irf8-GFP mice using anti-GFP antibody. Peritoneal macrophages from the same mice were also examined as a reference. We identified 16,935 IRF8 peaks in adult microglia and 12,062 in peripheral peritoneal macrophages (Fig. 1A). About half of the IRF8 peaks were in the intergenic regions with >5 kb distal from the genes, while about 20% were at the promoter regions in both cell types (Fig. 1A). *De novo* motif analysis revealed enrichment of PU.1/IRF composite motif (TTCC…G/CTTT) and classical ISRE (GAAAG, Fig. 1B). DEseq2-based statistics showed a differential IRF8 binding profile between microglia and peritoneal macrophages (Fig. 1C). Further, GREAT GO annotation indicated microglia specific IRF8 peaks (n=9,679) to be related to cell/leukocyte activation and differentiation (Fig. 1D; upper panel). In contrast, macrophage specific IRF8 peaks (n=2,251) were related to immune responses and inflammatory responses against pathogens (Fig. 1D; lower panel). In addition, IRF8 binding was found near microglia identity genes, such as Sall1 and Cx3cr1^11, 27^, but not in peritoneal macrophages (Fig. 1G).

IRF8 binding peaks on P9, P14, and adult microglia are shown in Fig. 1E. The IRF8 binding peaks on P9 microglia were very sparse. The binding increased at P14, although still less than on adult microglia, indicating a stepwise increase in IRF8 binding (Fig. 1E). Furthermore, cumulative distribution frequency graphs plotting the distance between two neighboring IRF8 binding sites (Fig. 1F, right) showed that the distance in the adult is the shortest, while it is longer in P14 microglia and peritoneal macrophages, and the longest in P9 microglia, which indicates that IRF8 on microglia could form arrays as illustrated in Fig. 1F (left). IGV tracks corroborated the stepwise increase in IRF8 binding, observed over the enhancer region of Sall1 and Cx3cr1 genes (Fig. 1G, left). These regions were not occupied by IRF8 in peritoneal macrophages (Fig. 1G, left). Rather, IRF8 was bound to regulatory regions of Clec4n, Lyz1/2, active in peritoneal macrophages (Fig. 1G, right). These results reveal that IRF8 binding to the microglial genome is developmentally controlled and reaches a maximum when microglia gain full functional activity. It is of note that despite the stepwise increase in binding activity, the amount of IRF8 was virtually the same during postnatal stages (Fig. S1B), suggesting a mechanism that directs IRF8’s ability to gain access to the microglial genome.

### IRF8 directs transcription cascades to activate microglia identity genes

**Figure 2:**
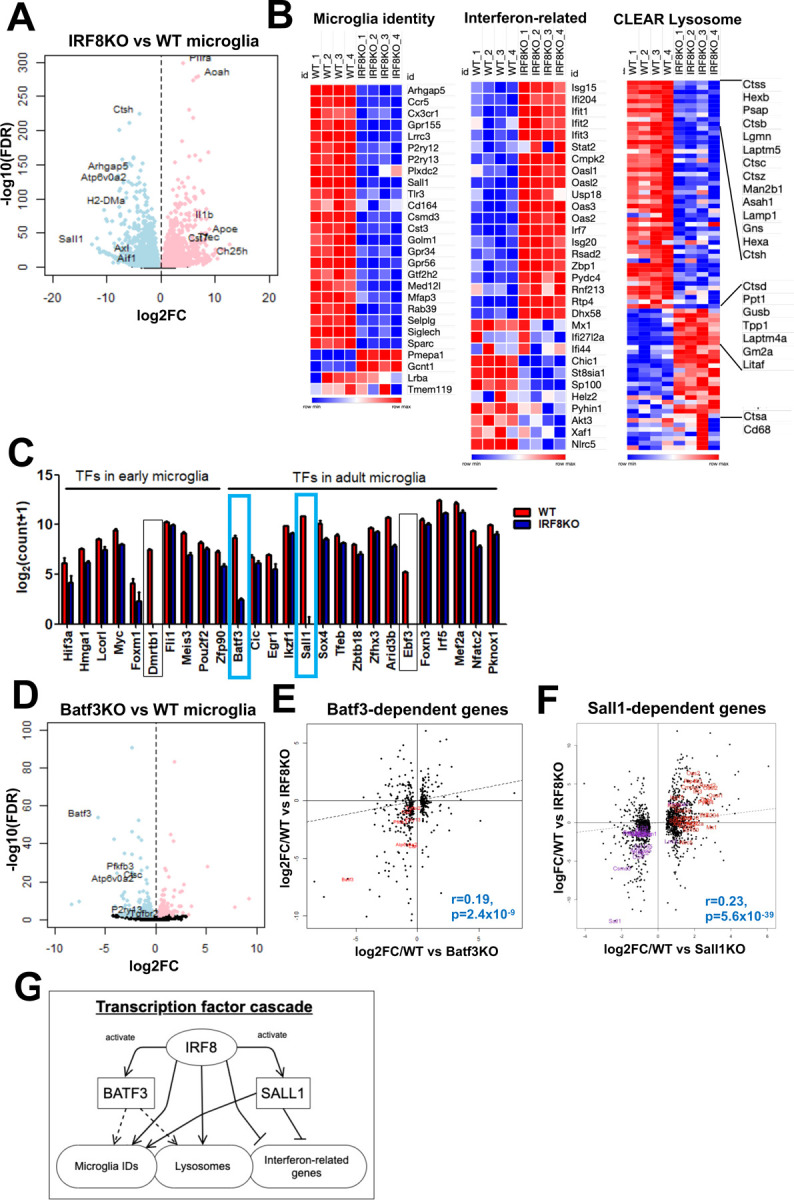
Identification of IRF8-driven transcription factor cascades in shaping microglia-specific transcriptional programs A. Volcano plot for RNA-seq comparing WT and IRF8KO microglia. Genes upregulated in IRF8KO microglia are indicated in pink (n=2,356), and genes downregulated in IRF8KO cells are in blue (n=2,682, FDR<0.01). Representative genes are shown with a label. B. Heatmaps for indicated gene sets. “Microglia identity” and “Interferon-related” gene sets were referred to as “cluster 6: Microglia” and “Interferon-Related” in the previous publication (ref.^11^). The CLEAR lysosome gene set was obtained from elsewhere (ref.^28^). C. Transcription factors downregulated in IRF8KO microglia (FDR<0.01 cut-off) are shown. A list of transcription factor genes was obtained from the previous publication (ref.^14^). Genes that show more than four log-scale reductions in IRF8KO cells are boxed, and Sall1 and Batf3 in the blue box were further investigated. D. Volcano plot for RNA-Seq data comparing WT and Batf3KO microglia. The genes upregulated in Batf3KO cells are shown in pink (n=172) and downregulated genes in blue (n=274, FDR<0.01). The representative genes are indicated in the plot. E. Correlation biplot showing that Batf3 dependent genes correlate with those dependent on IRF8. The DEGs from Batf3KO microglia RNA-Seq were analyzed with the Kendall rank correlation test (r=0.19, p=2.4×10^−9^). Microglia identity genes shared between Batf3KO DEGs and IRF8KO DEGs are labeled red. F. Correlation biplot showing that Sall1 dependent and IRF8 dependent genes overlap. All up and down DEGs from Sall1KO microglia RNA-Seq obtained from the previous publication (ref.^27^). were analyzed with the Kendall rank correlation test (r=0.23, p=5.6×10^−39^). The microglia identity genes shared between Sall1KO DEGs and IRF8KO DEGs are labeled purple, and those of interferon-related genes are red. G. A model illustrating IRF8 dependent transcription cascade. IRF8 induces expression of downstream TFs, BATF3 and SALL1. These TFs, along with IRF8, then activate indicated target genes. This dual cascade would cover all three gene sets, microglia identity, interferon related, and CLEAR lysosomal genes.

To study how IRF8 binding controls microglia’s transcriptional programs, we carried out bulk RNA-seq for microglia isolated from wild-type (WT) and IRF8KO mice. FACS analyses of brain cells revealed two CD45^+^ populations in the IRF8KO brain, one CD11b^high^CD45^+^ corresponding to WT microglia, and another CD11b^low^CD45^+^ subset, termed P3, which was absent in WT brain (Fig. S2A). The former subset expressed CX3CR1, a typical microglial marker, although its levels were lower than WT microglia. P3 subset present only in the IRF8KO brain did not express CX3CR1, instead expressed Ly6C, unraveling the presence of an extra cell population (Fig. S2B). We isolated microglia from WT and IRF8KO brains and processed them for microglial transcriptome analyses. Statistical analysis revealed a large number of genes that were up or downregulated in the absence of IRF8 (Fig. 2A). A set of microglia identity genes, e.g., P2ry12, Siglech, and Ccr5, encoding surface proteins and a transcription factor, Sall1, were drastically reduced in IRF8KO microglia (Fig. 2B left). In addition, many lysosomal genes represented by CLEAR genes were also reduced in IRF8KO cells, e.g., Ctsb, Ctsc, and Lamp1 (Fig. 2B right)^28^. Consistent with the data, Tfeb, a transcription factor that drives CLEAR lysosomal gene expression^28^, was reduced in IRF8KO microglia (Fig. 2C). On the other hand, a set of interferon related genes were aberrantly upregulated in IRF8KO cells, suggesting dysregulated host defense (Fig. 2B middle). In addition, many DAM/NDG genes were upregulated in IRF8KO microglia, including Apoe, a major AD risk factor^11,12,29,56,57^ (Fig. 7A, B).

Given that IRF8 activates downstream transcription factors to promote monocyte function, we examined levels of transcription factors expressed in postnatal microglia^14, 20, 30^. Among them, Batf3 and Sall1 were markedly downregulated in IRF8KO cells (Fig. 2C, blue square)^14^. BATF3, a basic leucine zipper motif (bZIP) family of a transcription factor, is known to be critical for generating a dendritic cell subset^31^. Because the role of BATF3 in microglial transcriptome remained unelucidated, we next analyzed RNA-Seq in Batf3KO and WT microglia (Fig. 2D). As anticipated, genes downregulated in Batf3KO microglia, such as P2ry13 and Ctsc were also downregulated in IRF8KO cells, indicating that IRF8 and BATF3 control an overlapping set of target genes. Consistent with this, BATF3-dependent genes showed a significant correlation with IRF8-dependent genes (Fig. 2E). Sall1 is a transcription factor of the Spalt family, expressed selectively in microglia. Buttgereit et al. reported that SALL1 is required for the expression of microglia signature^27^. The correlation biplot also showed a significant overlap between SALL1 dependent genes and IRF8 dependent genes, including microglia identity and Interferon related gene sets (Fig. 2F). Thus, IRF8 activates Sall1 expression, which in turn activates or represses genes that shape microglia signature. These data indicate that IRF8 creates transcription cascades and activates Sall1 and Batf3 expression, thereby inducing downstream target genes necessary for microglia identity and function (Fig. 2G).

### IRF8 configures microglia specific enhancers

**Figure 3:**
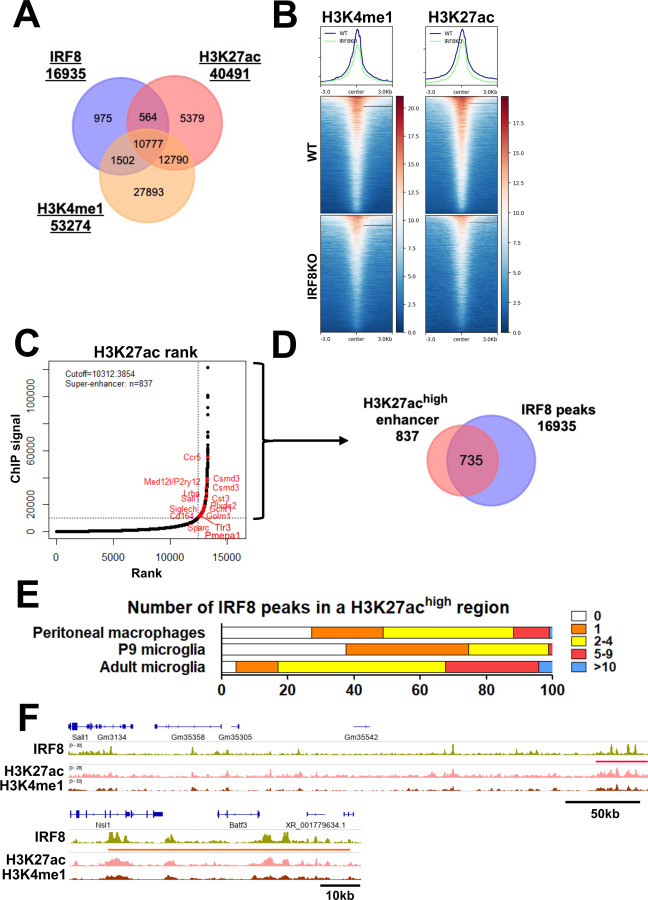
Adult IRF8 is an active enhancer that establishes an enhancer landscape in adult microglia A. A Venn diagram showing the overlap among IRF8 peaks, H3K4me1, and H3K27ac marks. B. Heatmaps showing H3K27ac (left) and H3K4me1 (right) signal intensity centered on IRF8 peaks ± 3.0kb in adult WT and IRF8KO microglia. Shown above heatmaps are average peak intensities. C. H3K27ac ranked super enhancers in adult WT microglia. IRF8 dependent genes neighboring super enhancers are indicated in red. D. Venn Diagram depicting the overlap of H3K27ac super enhancers and IRF8 peaks in adult WT microglia. E. IRF8 peak counts of adult microglia, P9 microglia, and peritoneal macrophages in the H3K27ac^high^ regions. F. Representative IGVs visualizing the distribution of IRF8 peaks, H3K4me1, and H3K27ac marks near/at Sall1 (top) and Batf3 genes (bottom). An H3K27ac^high^ region is marked by a red bar. IRF8 bound densely to the Batf3 regulatory region (orange bar), distinct from super enhancers. See also Fig.S3C–G for further description.

Above results established that IRF8 binding represents a critical basis of microglia’s transcriptional programs. To further characterize the chromatin environment in which IRF8 binds, we examined histone marks linked to active transcription, particularly those denoted for enhancers, H3K4me1 and H3K27ac^32, 33^. Of 16,935 IRF8 binding sites, about 73% and 66% of IRF8 colocalized with H3K4me1 and H3K27ac, respectively, and 64% of IRF8 binding sites carried both enhancer marks (Fig. 3A). Conversely, H3K4me1 and H3K27ac marks near IRF8 peaks were much lower in IRF8KO cells than in WT cells, indicating that IRF8 helps set an active chromatin environment in microglia (Fig. 3B). Differential deposition analysis for H3K27ac and H3K4me1 marks revealed that both WT and IRF8KO microglia exhibited cell type specific histone modification patterns (Fig. S3A). Since the H3K27ac is a surrogate marker for super-enhancers and super-enhancers neighbor many genes critical for defining cell type specificity and function^33, 34^, we examined super-enhancers using the ROSE program^33^. As expected, many IRF8-dependent microglial identity genes were neighbored by some of 837 super-enhancers, including Sall1, Siglech, and Cst3 (Fig. 3C). Indeed 735 of 837 (87.8%) super-enhancers were bound by IRF8 (Fig. 3D). While most H3K27ac^high^ regions were populated by IRF8 binding in adult microglia, this was not the case in early postnatal P9 microglia and peritoneal macrophages (Fig. 3E). In addition to super-enhancers, IRF8 was abundantly enriched in smaller enhancers found in specific intergenic and intronic regions overlapping with H3K4me1 (Fig. S3C–G). IGV tracks for the Sall1 and Batf3 genes show that clusters of IRF8 binding coincide with those of H3K27ac and H3K4me1 (Fig. 3F). These results indicate that IRF8 binding guides the formation of enhancers unique to microglia during postnatal development.

### IRF8 confers chromatin accessibility on postnatal microglia and sets DNA methylation patterns

**Figure 4:**
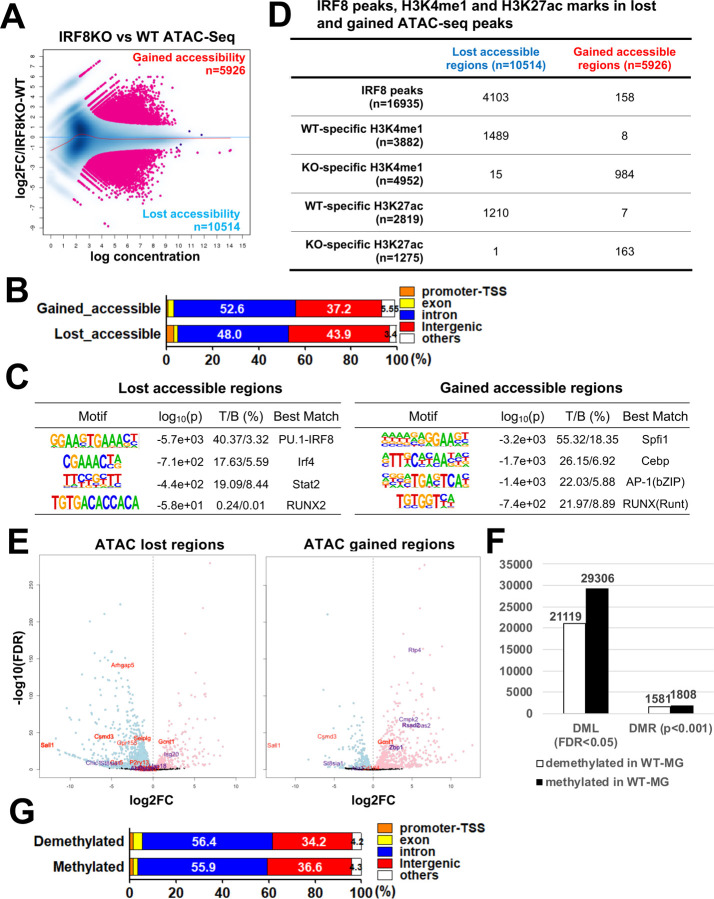

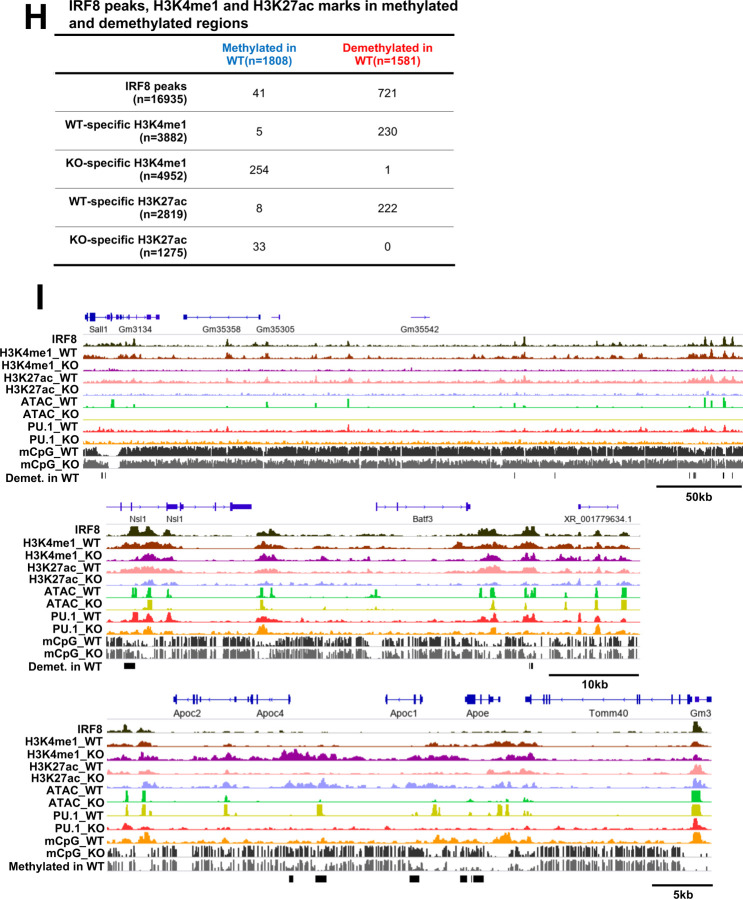
IRF8 confers chromatin accessibility on postnatal microglia and sets DNA methylation patterns A. MA plot showing ATAC-seq signals comparing WT and IRF8KO microglia (n=2). The differentially accessible regions (FDR<0.05) are colored in pink. The number of gained or lost accessible regions in IRF8KO microglia was shown at the corresponding corner of the area. B. The genome-wide distribution of gained and lost ATAC-seq peaks as determined by HOMER. C. HOMER *de novo* motif analysis for gained and lost ATAC-seq peaks. The top 4 motifs are shown. D. The number of IRF8, H3K27ac, and H3K4me1 peaks (WT or IRF8KO specific) overlapped with lost or gained ATAC-seq peaks. See Fig.S3A for the WT or IRF8KO specific peak identification. E. Volcano plot showing a correlation between ATAC-seq peaks and RNA-seq data. Microglia identity genes (representative “down” genes) and Interferon related genes (representative “up” genes) are labeled in red and purple, respectively. The nearest genes for gained or lost ATAC-seq peaks correlate with genes up or down-regulated in IRF8KO microglia. F. The number of differentially methylated single nucleotide loci (DML; FDR<0.05) and the differential regions (DMR; p<0.001). Numbers in the graph are differentially methylated and demethylated loci/regions in WT cells. G. Genome-wide distribution of the differentially demethylated (top) and methylated (bottom) regions identified with HOMER. H. Numbers of IRF8, H3K27ac, and H3K4me1 (WT or IRF8KO specific) peaks in differentially methylated or demethylated regions. I. Representative IGVs visualizing IRF8. H3K4me1, H3K27ac, ATAC-seq, PU.1 peaks and methylated DNA (CpG) status on Sall1 (top), Batf3 (middle), and Apoe (bottom) in WT and IRF8KO cells. Methylated and demethylated regions are indicated by black bars.

To investigate genomic sites accessible for transcription and whether IRF8 influences the process, we performed an assay for transposase-accessible chromatin (ATAC) sequencing^35^. Differential accessibility analysis revealed that 10,514 peaks were lost in IRF8KO microglia while gaining 5,926 new peaks (Fig. 4A). As anticipated, gained and lost peaks were mostly localized to intergenic regions and introns (Fig. 4B). *De novo* motif analysis showed that lost peaks were enriched with the PU.1/IRF composite motifs (Fig. 4C, left). Whereas, gained peaks were enriched with motifs for CEBP, AP-1, and RUNX binding sites (Fig. 4C, right). Further, ATAC peaks lost in IRF8KO cells were enriched with WT-specific H3K4me1 and H3K27ac marks (Fig. S3A, 4D). On the other hand, IRF8KO-specific H3K4me1 marks were found in gained ATAC peaks. Besides, IRF8KO cells had extra PU.1 binding sites absent in WT microglia (Fig. S3B). Comparison of ATAC-seq peaks and RNA-seq profiles found at lost ATAC-seq peaks in IRF8KO cells were enriched in genes downregulated in IRF8KO cells, such as microglia identity genes (Fig. 4E, in red). Whereas gained peaks were enriched in genes upregulated in IRF8KO cells (Fig. 4E, in purple). IGV tracks showed multiple ATAC peaks on and near the Sall1 and Batf3 genes in WT cells but few in IRF8KO cells (Fig. 4I). Further, in WT cells, H3K27ac and H3K4me1 marks were abundantly present at these ATAC-seq sites but reduced in IRF8KO cells (Fig. 4I). PU.1 binding sites also revealed a correlation with ATAC-seq peaks (Fig. 4I). However, Apoe, expressed in IRF8KO cells but not WT cells showed multiple ATAC-seq peaks in IRF8KO cells, not in WT cells. Together, these data indicate that IRF8 sets chromatin accessible sites in microglia, thereby establishing a microglia specific transcriptional program.

DNA methylation influences the epigenetic landscape in various somatic cells^36^. DNA methylation has been described on CpG-rich regions near the promoter, where methylated CpG correlates with transcriptional repression, while demethylated CpG with active transcription^37^.To examine whether IRF8 regulates DNA methylation in microglia, we performed whole genome bisulfite sequencing. We identified over 20,000 methylated loci (DML) that were differentially regulated in WT and IRF8KO microglia (Fig. 4F)^38, 39^. In addition, 1,808 DNA methylated regions (DMR) and 1,581 demethylated regions were differentially regulated in WT and IRF8KO cells, indicating that IRF8 takes part in DNA methylation in microglia (Fig. 4F). Both methylated and demethylated regions were in intergenic regions and introns, away from the proximal promoter and TSS (Fig. 4G). We found that IRF8 binding sites were located mostly in demethylated regions in WT cells (Fig. 4H, top). Further, methylated regions were enriched with the H3K4me1 and H3K27ac marks present in IRF8KO cells, while demethylated regions were enriched with those marks in WT cells (Fig. 4H). Correlation biplot analysis found that demethylated regions in WT cells highly correlated with ATAC-seq peaks present in WT cells, while methylated regions correlated with ATAC-seq peaks found in IRF8KO cells (Fig. S4A). These data indicate that demethylated regions were accessible to transcription factors, while methylated sites were not. Consistent with this idea, GO analysis revealed that demethylated regions were enriched with terms such as regulation of immune responses and leukocyte activity (Fig. S4B). In IGV tracks, individual methylated and demethylated sites are shown for Sall1, Batf3, and Apoe (Fig. 4I, bottom two lanes). Sall1 and Batf3 were enriched with demethylated sites in WT cells, but not IRF8KO cells. Conversely, Apoe exhibited many methylated sites in WT cells, but demethylated sites in IRF8KO cells. The above data provide the first evidence that IRF8 suppresses DNA methylation in gene specific manner in microglia. Together our results indicate that the regulation of DNA methylation is an integral mechanism for setting epigenomic landscape in microglia, which IRF8 dictates.

### Continuous IRF8 expression is required for the transcriptome in adult microglia

**Figure 5:**
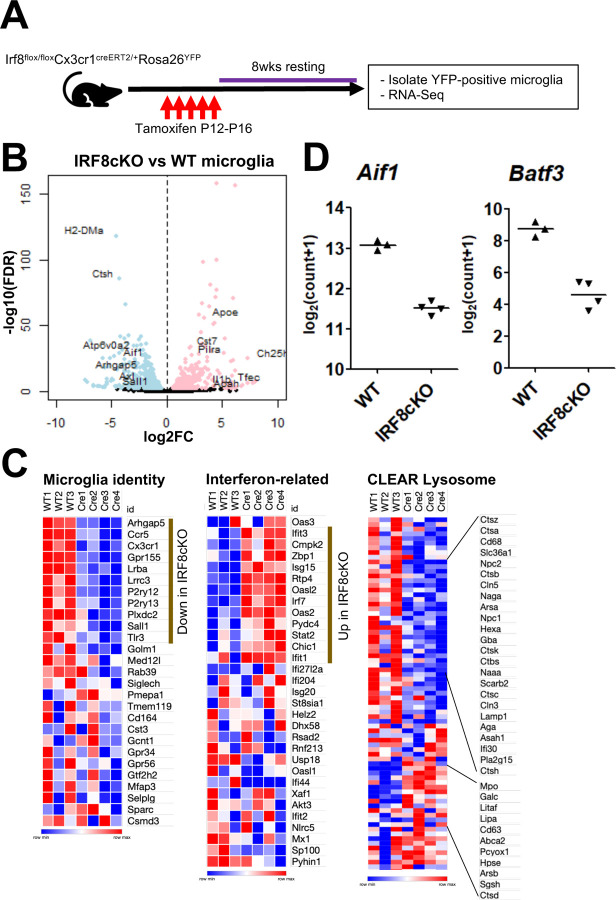
Postnatal deletion of Irf8 causes gene expression changes analogous to constitutive IRF8KO microglia A. The strategy for conditional Irf8 deletion in postnatal microglia. P12 or four weeks old Irf8^flox/flox^Cx3cr1Cre^ERT2/+^Rosa26^YFP^ mice were injected with Tamoxifen 5 times, followed by eight weeks of rest. Microglia were isolated and used for RNA-seq analysis. B. Volcano plot for transcriptome profiles of WT and IRF8cKO microglia. Up and down regulated genes in IRF8cKO microglia are shown in pink (n=334) and blue (n=546, FDR<0.01), respectively. Representative up and down regulated genes are indicated in the plot. C. Heatmaps comparing expression levels of the indicated gene sets between WT and IRF8cKO microglia. D. Expression of Aif1 (Iba1) and Batf3 genes in WT and IRF8cKO microglia. Each triangle represents logarithmized counts+1 from RNA-seq data of different biological replicates.

To assess whether IRF8 is required for postnatal microglia, we constructed Irf8^flox/flox^Cx3cr1cre^ERT2^ mice to allow for conditional Irf8 deletion. The mice also carried a Rosa26-loxP-stop-loxP-EYFP reporter, with which we could identify and isolate Cre-expressing microglia^40^ (Fig. 5A). To our surprise, the efficiency of Irf8 deletion was very poor (28% ± 18%SD, Fig. S5A) when Tamoxifen was administered on 4-week-old mice five consecutive times. To mitigate this problem, we resorted to deleting Irf8 in younger mice, between P12 and P16, and found a considerable improvement in Irf8 deletion efficiency (62% ± 5%SD, Fig. S5A). The basis of poor deletion efficiency is currently unclear, but it may be due to a shift in Cre accessibility.

RNA-seq was performed for EYFP positive microglia from IRF8cKO (Cx3cr1-Cre/wt) and WT microglia (Cx3cr1-wt/wt) treated with Tamoxifen (Fig. 5A). We found that 334 genes were downregulated, while 546 genes were upregulated in IRF8cKO cells relative to WT cells (Fig. 5B). Many DEGs found with IRF8cKO cells overlapped with those in constitutive IRF8KO cells (Fig. S5B). Namely, genes denoting microglia identity, such as P2ry12, Siglech, and Aif1 (Iba1), were markedly downregulated in IRF8cKO cells (Fig. 5C, 5D). Similarly, the expression of transcription factors, Sall1 and Batf3, IRF8’s downstream targets, and CLEAR Lysosome genes were much lower in IRF8cKO cells (Fig. 5C, 5D). Moreover, aberrant induction of DAM/NDG genes and Interferon-related genes was also observed in IRF8cKO microglia, mimicking changes in IRF8KO microglia (Fig. 5B, S7B). Together, these results show that continuous IRF8 expression is required for maintaining the transcriptional programs in postnatal microglia.

### Single-cell RNA-seq analysis of microglia reveals a progenitor-like population

**Figure 6:**
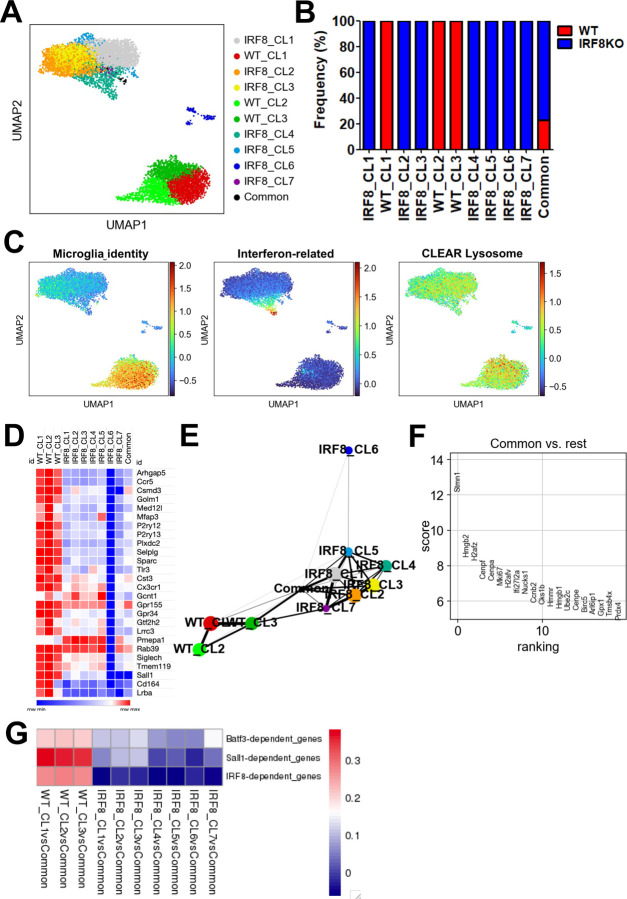
scRNA-seq analysis reveals transcriptome heterogeneity and the presence of a progenitor like population A. UMAP derived from WT and IRF8KO microglia aggregates. Leiden clusters were colored and labeled as indicated. The data were obtained from the pool of three individual WT and IRF8KO microglia preparations. B. Percentages of WT and IRF8KO genotypes in indicated clusters. C. UMAP presentation of indicated gene sets (compare with heatmaps in Fig. 2B). D. Gene expression heatmap showing the expression of microglia identity gene set in each WT and IRF8KO cluster. Note that expression of all identity genes is absent in IRF8KO Cluster 6. E. PAGA network depicted with the default parameters. Each node indicates the corresponding Leiden cluster. F. Rank plot aligning the top 20 genes by scoring expression level. The genes in the Common cluster were compared to those of all the other clusters. G. Heatmap displaying the correlation coefficients between Common cluster and other clusters for IRF8, BATF3, or SALL1 dependent gene sets.

Next, we performed single-cell RNA-Seq (scRNA-Seq) analysis to assess transcriptome diversity. After filtration, 3,504 WT and 5,303 IRF8KO cells expressing a total of 15,376 genes were selected. Leiden clustering denoted three WT clusters, seven IRF8KO clusters, and a “Common” cluster consisting of WT and IRF8KO cells (Fig. 6A and B). The SigEMD pipeline identified 6,149 DEGs between WT and IRF8KO cells, which covered 62% (3016/4848) of DEGs found in bulk RNA-seq analysis, lending credence to our data (Fig. S6B). Microglia identity gene expression was higher in all WT clusters than in all IRF8KO clusters (Fig. 6C, left). Population heterogeneity was evident within IRF8KO cells, in that interferon-related genes were exclusively found in Clusters 4 (Fig. 6C, middle). In line with this, GO analysis of this Cluster ranked interferon-related terms as the top categories (Fig. S6C). Unlike this, CLEAR lysosomal gene expression was uniformly decreased in all IRF8KO cells (Fig. 6C, right). Notably, IRF8KO Cluster 6 did not express microglia identity genes, nor other genes expressed in IRF8KO cells. Instead it expressed Ly6c2, encoding Ly6C (Fig. 6C, S6A). Thus, this cluster likely represents the P3 population in IRF8KO brain (Fig. S2B). The Common cluster exhibited unusual features, in that most microglial genes were absent in this population (Fig. 6D). This raised the possibility that Common cluster represents a population prior to microglia maturation. To delineate a relationship between Common cluster and other clusters, we applied the PAGA program, which provided a trajectory network (Fig. 6E)^41^. This network illustrated a tight connection with the Common cluster to all WT and IRF8KO clusters except IRF8KO Cluster 6. Another unusual feature of Common cluster was the expression of cell cycle genes such as Cenpa, Ccnb2, and Mki67, a marker for cell proliferation (Fig. 6F). If Common cluster represents a developmentally immature population, it would predict that IRF8 dependent transcription cascade would be present only when it differentiates into WT clusters. To test this, we examined genes downstream of IRF8, BATF3, or SALL1 in WT and IRF8KO clusters relative to Common cluster. Correlation analyses revealed a significant correlation only in WT clusters, not any IRF8KO clusters, as predicted. Together, these results support the idea that Common cluster represents a predecessor of microglia, possessing the capability to develop into functioning microglia.

### Irf8 deletion ameliorates AD pathology in the mouse model

**Figure 7:**
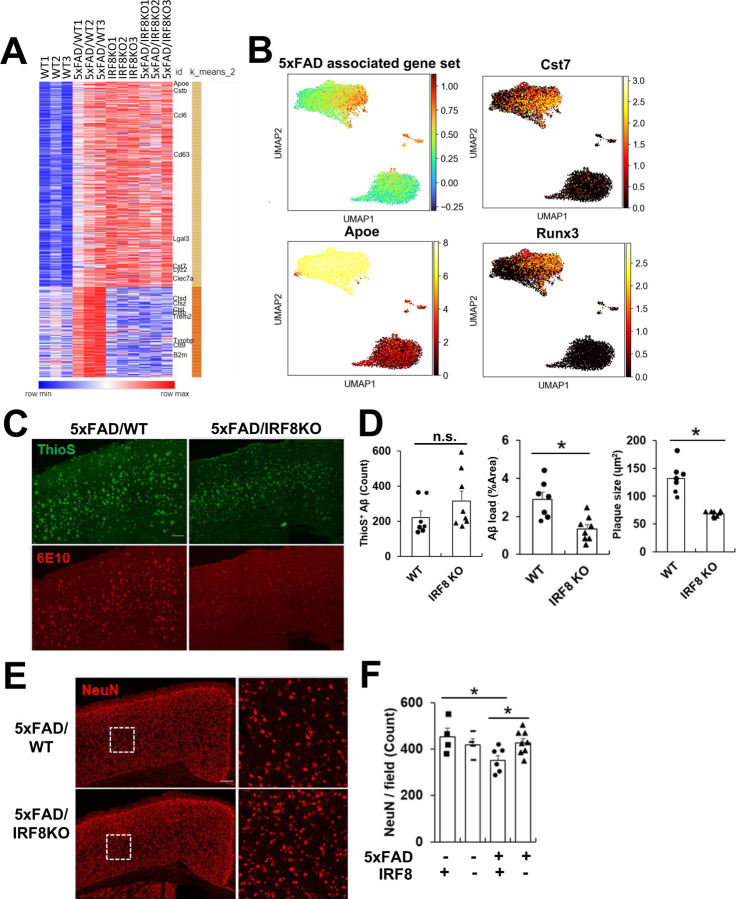
IRF8KO microglia lessens AD pathology in the 5xFAD mouse model, despite spontaneous DAM/NDG gene induction A. Heatmap showing the 5xFAD associated gene set expression in WT, 5xFAD/WT, IRF8KO, and 5xFAD/IRF8KO microglia. Data are from RNA seq analysis of microglia from nine months old mice. Data were clustered by K-mean methodology (k=2), and representative genes were labeled on the right. B. UMAP presentation of 5xFAD associated gene set and its representatives, Apoe and Cst7, from the scRNA-seq analysis. Runx3 is a transcription factor exclusively expressed in IRF8 Cluster1, indicating an association of RUNX3 with the transcriptome of this cluster. C. Representative histology images showing core-dense plaques in the cortex region of a year-old 5xFAD/WT or 5xFAD/IRF8KO brain. Brain sections were stained with Thioflavin S (green) and 6E10 antibody (red). Scale bar; 200 μm. D. Quantification of the number of ThioS+ aggregates (left), amount of Aβ deposition (middle), and size of Aβ plaques (right). Values represent the average of five fields from each brain. Data: Mean +/− SEM; *: p-value <0.05. E. Representative histology images of NeuN staining in the cortex area of a year-old 5xFAD/WT or 5xFAD/IRF8KO brain. Scale bar; 200 μm. On the right are enlarged images from the bracketed regions on the left. F. Quantification of NeuN positive cells. Values are the average of five fields from three brains. *: p<0.05.

IRF8 has been shown to promote neuroinflammation and implicated in AD pathogenesis in mice^21, 25,42, 43^. To further address the role of IRF8 in AD, we first compared microglia transcriptome profiles of WT and 5xFAD mice at 9 months of age^44^. We found 699 genes upregulated in 5xFAD microglia over WT cells, which we regarded as the 5xFAD associated gene set. This gene set contained many DAM/NDG genes described earlier, such as Apoe, Tyrobp, and Cst7 (Fig. S7A)^10,11^. Interestingly, many genes in this gene set, mostly DAM/NDG genes in an early stage of human and mouse AD microglia^29, 43, 45^, were also upregulated in IRF8KO microglia without the 5xFAD background (Fig. 7A). Further, microglia from 5xFAD/IRF8KO mice showed essentially the same expression pattern as IRF8KO microglia (Fig. 7A). These DAM/NDG genes were upregulated in IRF8cKO microglia even from much younger mice (three months old, Fig. S7B, S7C). Thus, Irf8 deficiency leads to the induction of DAM/NDG genes. In addition, scRNA-seq analysis found that DAM/NDG gene expression was most prominent in Cluster 1 of IRF8KO cells (Fig. 7B). Transcription factor Runx3 was also upregulated in this cluster (Fig. 7B).

To further examine the role of IRF8 in AD, histological analyses were performed with a year-old 5xFAD/WT and 5xFAD/IRF8KO brains using Thioflavin S (ThioS) and 6E10 antibody^46^. The former detects polymerized Aβ sheets, while the latter stains both polymerized and unpolymerized Aβ. Confocal images revealed that the number of ThioS positive aggregates was similar between 5xFAD/WT and 5xFAD/IRF8KO brains. However, the amyloid load and plaque size were smaller in the 5xFAD/IRF8KO than in the 5xFAD/WT brain (Fig. 7C, quantification in 7D). Moreover, in the 5xFAD/IRF8KO brain, many of the Aβ signals detected by 6E10 (green) did not directly colocalize with Iba1, a marker for microglia (Fig. S7D, red), whereas extensive colocalization was evident in WT cells. These results suggest that 5xFAD/IRF8KO microglia are defective in the recognition and perhaps internalization of Aβ in line with the report^42^. We next sought to assess the effect of Irf8 deletion on overall neuronal health in the 5xFAD brain. We thus stained cortical regions of a year-old 5xFAD/WT and 5xFAD/IRF8KO brains with NeuN antibody to detect intact neurons^54^. NeuN immunoreactivity is lower than those from 5xFAD/IRF8KO brains than the 5xFAD/WT brains (Fig. 7E, quantification in 7F). These results indicate that deletion of IRF8 lessens neuronal damage in the 5xFAD brain.

To further assess the role of IRF8 in AD, we checked Axl expression, as AXL is reported to regulate microglia’s ability to phagocytose Aβ^47^. Axl levels were lower in IRF8KO microglia than in WT microglia with and without 5xFAD background (Fig. 7A, S7F). These results suggest that IRF8 would affect AD pathology in a complex manner, inhibiting Aβ spread on one hand, and facilitating phagocytic clearance of Aβ on the other.

## Discussion

**Figure 8:**
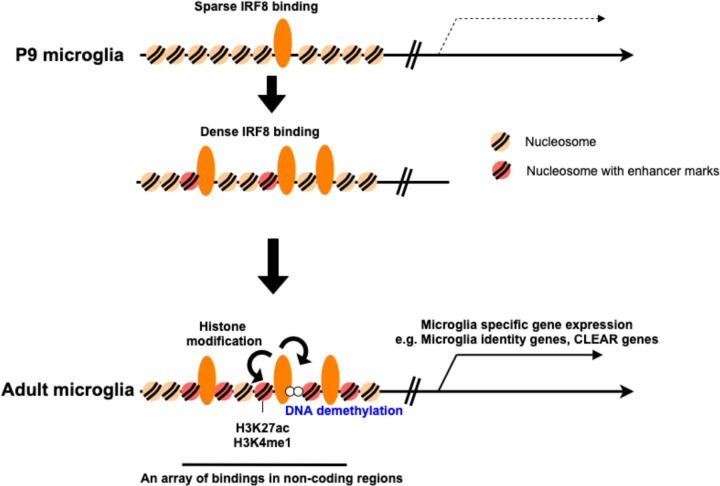
Model for the role of IRF8 in postnatal microglia development At the early postnatal stage (P9 microglia), IRF8 binding to the microglia genome is sparse (Top). After P14 through adulthood, IRF8 binding becomes denser, forming arrays along with histone modifications (Middle). Full IRF8 binding leads to the formation of microglia specific enhancers (large and small), opens relevant regions of chromatin and stably demethylates enhancer regions of DNA (bottom), which, taken together, establishes transcriptional programs in microglia.

This study aimed at elucidating the mechanism of postnatal microglia development and the role IRF8 plays in the process, the subject that has long remained elusive. It was critical first to determine genome-wide IRF8 binding sites and epigenome structures in the developing microglia. We show that IRF8 binding occurs in a characteristic stepwise fashion. IRF8 gained binding activity gradually, from P9 through P14. Binding was sparse on P9, increased on P14, and reached the maximum in adults. Microglia from P9 to P14 are still immature, gaining full identity and functionality later^17^. Thus, full IRF8 binding coincided with the acquisition of microglia identity and functionality. It was striking that a stepwise increase in IRF8 binding was not due to corresponding increase in IRF8 expression, since IRF8 levels were constant during this period and through adulthood, suggesting an epigenome-based mechanism, yet to be deciphered. In adult microglia, IRF8 peaks were often in dense repeats and within or near H3K27ac and H3K4me1 peaks, indicating that IRF8 binds mostly in the enhancers, both large and small. Furthermore, IRF8 binding was obligatory for installing chromatin accessibility sites, as revealed by ATAC-seq analysis. Many ATAC-seq peaks were in the IRF8 dependent enhancers, as exemplified by those for Sall1 and Batf3, IRF8 dependent transcription factors endowing microglia’s transcriptome profiles and phenotypes. These ATAC-seq peaks were lost in IRF8KO microglia, which in turn, gained alternate ATAC-seq peaks neighboring genes upregulated in IRF8KO microglia, including DAM/NDG genes, such as Apoe. We further show, by genome-wide bisulfite sequencing, that IRF8 sets DNA methylation patterns specific for microglia. The methylation patterns correlated well with the ATAC-seq peak patterns. Thus, Sall1 and Batf3 enhancers had unmethylated DNA. Conversely, in IRF8KO microglia, these regions of DNA were methylated, and the Apoe region was unmethylated. Together, the formation of enhances, chromatin opening, and DNA methylation patterns unique to microglia depend on developmentally regulated IRF8 binding. Thus, we suggest a model in which IRF8 binding is the primary initiating event that confers microglia specific epigenomic landscape (Fig. 8). Our data on IRF8cKO microglia further support this model, in that deletion of Irf8 from adult microglia led to the loss of microglia identity and function closely resembling the phenotype of microglia from constitutive IRF8KO mice, demonstrating that continuous IRF8 occupancy is necessary to maintain microglia specific chromatin and DNA methylation landscape.

Aside from this, it is noteworthy that Sall1 and Batf3, transcription factors that provide actual functionalities to microglia, are downstream of IRF8. Clearly, IRF8 forges transcription factor cascades to achieve microglia specific transcriptome programs. IRF8 promotes the differentiation of monocytes and dendritic cell subsets by activating downstream transcription factors^18,19^. These features of IRF8 are somewhat reminiscent of the “pioneer transcription factor” that enables other transcription factors to gain access to the proper DNA/chromatin sites, thus establishing functional regulatory domains^48^

scRNA-seq data on the Common cluster are relevant to the development and lifespan of microglia. This cluster, found in WT and IRF8KO cells, expressed multiple cell cycle and replication genes, which were not detected in other clusters. PAGA trajectory pointed out that this cluster is related independently to other clusters, possibly representing a predecessor population capable of differentiation into mature microglia. With a progenitor-like property, this population may be the source of postnatal and adult microglia, which replenishes microglia continually for life.

Irf8 deletion led to a strong reduction of the microglia identity gene set but unleashed marked expression of DAM/NDG and Interferon-related gene sets. Both gene sets are associated with AD and other neurodegenerative diseases^10, 11^. Nevertheless, AD pathology in 5xFAD brains without IRF8 was less severe than that with IRF8, as evidenced by reduced large Aβ plaques and enhanced NeuN immunoreactivity. Microglia are known to constantly survey the brain space, recognize Aβ and distribute them to other brain regions^42^. IRF8KO microglia are deficient in these activities, since they lack the ability to scan the brain to recognize Aβ, as verified by the absence of colocalization of Aβ with microglia. d’Errico et al. further reported that Aβ plaques did not spread in the IRF8KO brain relative to WT counterparts^42^. Pertinent to this, it has been shown that depletion of microglia by CSF1R inhibitors reduces plaque formation and neuronal loss^49^. Notwithstanding these results, IRF8 likely exerts a complex role, positive and negative, in AD progression, given that Axl and CLEAR/lysosomal genes, crucial for controlling AD, require IRF8 for full expression.

In summary, this study demonstrates the essential requirement of IRF8 for the adult development of microglia and its complex role in modulating AD progression.

## Online Methods

### Mice

B6(Cg)-*Irf8*^tm1.2Hm^/J (IRF8KO), B6.Cg-*Irf8*^tm2.1Hm^/J (Irf8-GFP), B6.Cg-*Irf8*^tm1.1Hm^/J (Irf8flox) and control wild-type C57BL/6J mice were maintained in the NICHD animal facility^25^. B6.129S(C)-*Batf3*^*tm1Kmm*^/J (Batf3KO), B6.129P2(Cg)-*Cx3cr1*^tm1Litt^/J (Cx3cr1^GFP^), B6.129P2(C)-Cx3cr1^tm2.1(cre/ERT2)Jung^/J (Cx3cr1cre^ERT2^), and B6SJL-Tg^(APPSwFlLon, PSEN1*M146L*L286V)6799Vas/^Mmjax (5xFAD) mice were purchased from Jackson Laboratory. Cx3cr1^GFP^ mice were crossed with IRF8KO mice. 5xFAD mice were backcrossed to C57BL/6J strain for at least seven generations and maintained hemizygote. To achieve microglia specific Irf8 depletion (IRF8cKO), we crossed Irf8flox mice with Cx3cr1cre^ERT2^ mice. Mice were given 300μg of Tamoxifen (Sigma) for five consecutive days at P12 or 2mg × 5 days at P28, and the Cre recombinase expression in microglia was confirmed using Rosa26^LSL-EYFP^ reporter mice. Female mice were used in this study unless specified, and all animal experiments were performed according to the animal study (ASP#17–044 and 20–044) approved by the Animal Care and Use Committees of NICHD, NIH.

### Microglia preparation

Mice were euthanized by asphyxiating with CO_2_ and transcardially perfused with 10 mL of ice-cold PBS. The brain was harvested, chopped with a single-edge razor blade, and ground in ice-cold Hanks-balanced salt solution using a 7ml Dounce homogenizer, then filtered through a 70μm cell strainer. The cells were resuspended in 30% isotonic Percoll and spun down 800xg for 30 min. The floating myelin layer was removed, and the cell pellet was further sorted by FACS AriaIIIμ (BD bioscience) after antibody staining. All reagents and instruments were kept in our 4°C cold room, and all procedures were performed at less than 4°C to avoid extra activation. For FACS cell sorting, the following antibodies were used: CD11b-Brilliant Violet 421 (CD11b-BV421, clone: M1/70, Biolegend), CD45-APC (clone: 30-F11, Invitrogen). The dead cells were gated out with staining of 7-Aminoactinomycin D (7-AAD; BD pharmingen, 1:100). We used CX3CR1-PE (FAB5825P, R&D), Ly6C-APCCy7 (clone:HK1.4, Biolegend), and F4/80-PECy7 (clone:BM8, Biolegend) antibodies for the expression analysis and reanalyzed the data with FlowJo v.7.6.5 software.

### Immunohistochemistry

Mice were anesthetized with 125mg/kg Ketamine and 10mg/kg Xylazine cocktail intraperitoneal injection. The brain was fixed by the transcardial perfusion of 4% PFA/PBS, followed by overnight incubation in an additional 4% PFA/PBS. After removing the fixative, the brain was cryoprotected with a 10–30% sucrose solution. The section was made with 30μm slices. Blocking was achieved with 4% BSA in PBS and 0.3% Triton X-100. Primary antibodies and working dilution are the following: Mouse anti-β-Amyloid antibody (1:500, clone: 6E10, Biolegend), Rabbit anti-NeuN monoclonal antibody (clone: A60, Millipore), and Rabbit anti-Iba1 antibody (Wako). The tissue was then incubated with the following fluorescence-labeled secondary antibodies; Alexa Fluor 488 Goat anti-mouse IgG (H+L) (Invitrogen), Alexa Fluor 633 Goat anti-mouse IgG (H+L) (Invitrogen), Alexa Fluor 633 Goat anti-rabbit IgG (H+L) (Invitrogen). The core-dense amyloid plaques were stained with 0.5% Thioflavin S (Sigma, T1892). The nuclei were stained with Hoechst33342 dye. The images were taken on a Zeiss AiryScan 880 confocal microscope and further analyzed with ImageJ/Fiji^50^. Five fields per section were randomly chosen in the cortical layer V and analyzed for quantification. The average value of 5 fields was presented for each mouse.

### Bulk RNA-Seq

Fifteen thousand cells were sorted out into TRIzol and stored at −70°C until library preparation. Total RNA was purified by phase extraction and processed to cDNA using SMART-Seq v4 Ultra Low Input RNA Kit (Takara bio USA, CA) with 15-cycle amplification. Adaptors were added to 1ng cDNA using the Nextera XT library prep kit (Illumina, CA). The libraries were sequenced on Nextseq500 with 55bp paired end read. For sequence data analysis, adapter sequences were trimmed with Trimmomatic v0.33 after the initial quality check, and then data was aligned with STAR v2.5.2b. Sequence duplicates were removed using Picard v2.17.11. Aligned tags were counted with Subread featureCounts, and differentially expressed genes were estimated using DESeq2^51^ with FDR<0.01. Batch effects were normalized at this point if needed. Heatmaps were depicted with Morpheus (https://software.broadinstitute.org/morpheus).

### Single-cell RNA-seq

Ten thousand cells were sorted out from 3 mice pools and immediately emulsified using a Chromium kit and 10x Genomics as the manufacturer’s protocol. The libraries were made using SMART-Seq v4 Ultra Low Input RNA Kit followed by Nextera XT library prep kit and sequenced sequenced 26bp for R1 and 98bp for R2 on HiSeq2000. The fastq files were first processed with Cellranger v3.1 pipeline to make gene expression matrices, and the following analyses were performed with Scanpy^52^. Cells with less than 500 or greater than 6,000 detected genes were filtered from analysis. Genes detected in less than three cells were also removed from the data. The feature counts were normalized to 10,000 reads per cell and logarithmized. The data were regressed out based on the highly variable genes marked with *min_mean=0.0125, max_mean=3, min_disp=0.5* options, and the cells exhibiting high standard deviation (SD>15) were excluded from the analysis. UMAP dimension reduction and Leiden clustering were employed to visualize the data with *n_neighbors=80, n_pcs=15,* and *min_dist=1.0* parameters. Each cluster’s connectivity was computed using PAGA^41^ with the default parameters.

### CUT&RUN

CUT&RUN methodology^53^ was used with some modifications. Protein A-bound micrococcal nuclease (pA-MNase) was kindly provided by Steven Henikoff. For histone marks, live microglial cells were sorted out and washed once with HEPES-NaCl buffer (HEN buffer; 20mM HEPES pH7.5, 150mM NaCl, 500μM Spermidine, Protease inhibitors cocktail), then counted. Ten thousand cells were immobilized to activated BioMag^®^Plus Concanavalin A (ConA)-beads (Bangs Laboratories, IN) in the presence of Mn^2+^ and Ca^2+^ ions (0.1mM MnCl_2_, 0.1mM CaCl_2_) at room temperature. The beads complex was then permeabilized using 0.005% Digitonin (Merck)-containing HEN buffer (Digitonin buffer) and incubated for 12hrs at 4°C with 0.25μg of the following antibodies in the presence of 2mM EDTA: rabbit anti-H3K27ac (ab4729, Abcam), rabbit anti-H3K4me1 (ab8895, Abcam). After antibody binding, beads were washed twice with Digitonin buffer and bound pA-MNase. After the washout of extra pA-MNase, beads-immunocomplex was activated in the presence of 3mM CaCl_2_ at 0°C for 30 min. The digestion was stopped by adding 10x Stop buffer (1.70M NaCl, 100mM EDTA, 20mM EGTA, 0.25mg/ml Glycogen, 0,25μg/μl RNaseA) and 15pg MNase-digested yeast DNA spike-in control. The nuclei were digested at 56°C for five hours after adding 40μg of Proteinase K and SDS at the final concentration of 0.1%. The immunoprecipitated DNA was purified by phase separation and removed undigested DNA with a half volume of AMPure beads. For library preparation, purified DNA was processed using SMARTer^®^ ThruPLEX DNA-Seq Kit (TAKARA Bio USA, CA) as the manufacturer’s protocol except for PCR cycles to avoid amplifying long fragments (13 cycles, 10s for extension). For transcription factors, the antibody-labeled brain homogenate was fixed with 1% Paraformaldehyde-PBS at room temperature for 10min right before cell sorting. Sorted cells were immediately lysed with nuclear extraction buffer (20mM HEPES pH7.5, 10mM KCl, 500μM Spermidine, 0.1% Triton X-100, 20% Glycerol, Protease Inhibitor cocktail) and counted. Thirty-thousand nuclei were immobilized with ConA-beads in the same nuclear extraction buffer and blocked using Wash buffer (20mM HEPES pH7.5, 150mM NaCl, 500μM Spermidine, 0.1% Bovine Serum Albumin, 1% Triton X-100, 0.05% SDS, Protease inhibitor cocktail) containing 4mM EDTA. The nuclei were incubated with a rabbit anti-GFP antibody (0.5μg/reaction, ab290, Abcam) or a rabbit anti-mouse PU.1 (EPR22624–20, Abcam) for 12hrs at 4°C. After binding to pA-MNase in Wash buffer, DNA was cleaved by adding 3mM CaCl_2_ for 30min at 0°C. MNase was inactivated by adding chelators mixture at the final concentration of 170mM NaCl, 10mM EDTA, 2mM EGTA, 0.005% NP-40, 25μg/ml Glycogen, and 15pg yeast spike-in control. Total DNA was extracted, and de-crosslinked by incubating overnight at 65°C in the high-salt buffer containing 0.1% SDS, 0.5mg/ml Proteinase K and 0.1mg/mL RNase A. The undigested DNA was removed as above, and the small fragments for sequencing libraries were harvested. The final libraries were purified by AMPure beads and sequenced with a 30bp paired end read. For data analysis, the adapter and low-quality sequences were removed from raw fastq data using TrimGalore. Then the data was aligned on mm10 mouse genome using Bowtie2 with *--local –very-sensitive-local --no-unal --no-mixed --no-discordant --phred33 -I 10 -X 700 –no-overlap --no-dovetail* options. Peak calling was performed by MACS2 with *-broad --keep-dup all -p 0.01 --min-length 1000* option for a broad histone mark, *--keep-dup all -q 0.01 –max-gap 640* option for a narrow histone mark and IRF8^54^. For PU.1, we employed *-q 0.05* instead because it is more consistent with the previous dataset (GSM1533906)^16^. The peak co-occurrence, genomic position, and motif analysis were performed using HOMER^32^ with *-d given* option. To determine highly concordant peaks across biological replicates, we employed “the Majority rule,” which picks up only peaks found within more than 50% of replicates^55^. The differential peaks were obtained using Diffbind-DESeq2^56^ with FDR<0.05 cut-off. Then all bam files were merged and used for IGV visualization and depicting average plots and heatmaps with Deeptools^57^. H3K27ac^high^ large regions were identified as “super-enhancer regions” of the ROSE’s readouts with default parameters^33^.

### Bulk ATAC-Seq

ATAC-Seq libraries were constructed as described before^35^. Fifty thousand isolated microglia were lysed in lysis buffer (10mM Tris-HCl pH7.4, 10mM NaCl, 3mM MgCl_2_, 0.1% NP-40). Following lysis, nuclei were tagmented using the Nextera DNA library prep kit (Illumina) for 30 minutes. Then DNA was purified and amplified for 12 cycles. The libraries were sequenced with a 50bp paired end read. After removing the adapter and low-quality sequences, the data were aligned to the mm10 genome with Bowtie2. Peaks were called on aligned sequences using MACS2 with *-p 0.01 --nomodel --shift -37 --extsize 73 -B --SPMR --keep-dup* all options. Peaks were then evaluated with less than 0.01 irreversible differential rates. Differentially accessible regions were identified using Diffbind-EdgeR with FDR<0.05^56^. Deeptools and HOMER were employed for further analyses, including motif enrichment analysis with the indicated options.

### Whole-genome bisulfite sequence

Genomic DNA was extracted from 40,000 male microglial cells using Quick-DNA Microprep plus kit (Zymo Research) as the manufacturer’s protocol. Then 50ng DNA was bisulfite-converted and processed to the library using Pico Methyl-Seq Library Prep Kit (Zymo Research), with 8-cycle PCR amplification. The libraries were sequenced with 75bp paired end read on HiSeq3000 in the NHLBI sequencing core (9.2x coverage on average). Based on the Bismark M-bias plot, the adapters, low-quality sequence, and an additional 10bp of 5’-end and 2bp of 3’-end were removed from the sequencing data using TrimGalore. The data were first aligned using Bismark^38^ with *-q --score-min L,0,-0.4 –ignor-equals --no-mixed --no-discordant --dovetail --maxins* options, and then unmapped sequences were aligned additionally with single-end mode. After deduplication and methylation calls, both files were merged into a single coverage file. Differential analyses were performed using R/DSS package^39^ using FDR<0.05 for the differentially methylated loci (DMLs) and p<0.001 for the regions (DMRs).

## Supplementary Material

Supplement 1

## Data Availability

All high-throughput sequence datasets generated in this paper are available in GSE231406. Some gene sets were obtained from publications as denoted individually.

## Abbreviations:

AD: Alzheimer’s disease
DAM/NDG: Alzheimer’s disease associated and neurodegeneration associated genes
DEG: differentially expressed gene
FACS: fluorescence-activated cell sorting
FC: fold change
FDR: false discovery rate
GFP: green fluorescent protein
GO: gene ontology
IRF8: interferon regulatory factor 8
TF: transcription factor
TES: transcription end sites
TSS: transcription start sites
YFP: yellow fluorescent protein
WT: wild type
