# Supplementary material for: IRF8 configures enhancer landscape in postnatal microglia and directs microglia specific transcriptional programs
Source: bioRxiv. 2023 Aug 17:2023.06.25.546453. Preprint. [Version 2] doi: 10.1101/2023.06.25.546453 (PMC10461927; doi:10.1101/2023.06.25.546453)
Supplement: Supplementary file 1 [file media-1.pdf]

Supplemental Figure Legend

Supplemental Figure 1

A. Gating strategy for the isolation of microglia and peritoneal macrophages. Cells were gated with $CD11b^{+}CD45^{low}$ for microglia and $CD11b^{+}F4/80^{+}$ for peritoneal macrophages. Staining with 7-AAD was performed when the live cells were needed.
B. IRF8 expression in microglia and peritoneal macrophages. Flow cytometry was performed to detect GFP for cells from IRF8-GFP/+ hemizygous transgenic mice (filled in magenta) and WT control (black line). The GFP expression in P9 microglia and P14 microglia of IRF8-GFP/GFP mice are also shown on the right.

Supplemental Figure 2

A. Flow cytometry of 7-AAD-negative brain cells stained with CD11b and CD45 antibodies from WT and IRF8KO mice. The median ratio of each subset from 7-AAD-negative cells was shown.
B. Flow cytometry profiles of CX3CR1, Ly6C, and F4/80 expression (filled in magenta) and isotype IgG (black line) in microglia from WT and IRF8KO brain or P3 population from IRF8KO brain.

Supplemental Figure 3 (also see Method)

A. MA plots showing H3K27ac (left) and H3K4me1 (right) histone marks comparing WT and IRF8KO microglia (n=2). The differential regions with FDR<0.05 are colored in pink.
B. MA plots showing PU.1 peak intensity comparing WT and IRF8KO microglia (n=2). The differential regions with FDR<0.05 are colored in pink.
C. The distance between two neighboring IRF8 peaks within an H3K27ac high region: Median;11.418 kb, 75% Quantile; 38.977 kb.
D. Venn diagram showing the overlap between $H3K27ac^{high}$ regions and regions occupied with an array of enhancer IRF8 peaks. To define an array of enhancer IRF8 peak regions, H3K4me1 marked IRF8 peaks within 40kb intervals were concatenated by ROSE. IRF8 also forms an array of bindings outside

H3K27ac^{high} regions (IRF8nSEC). See more details in the Supplemental methods below.

E. Genomic size of H3K27ac^{high} regions (super enhancers) and IRF8nSEC regions (smaller enhancers).
F. Genome wide distribution of super enhancers and IRF8nSEC region determined with HOMER.
G. Representative IGVs. Orange bar; IRF8nSEC region (smaller enhancers).

Supplemental Figure 4

A. Correlation biplot showing ATAC signal intensity and DNA methylation levels in 1,337 regions where all differential ATAC peaks and DMRs overlapped. Analyzed with the Kendall rank correlation test (coefficient r=-0.75, p=3.28x10^{-229}).
B. Gene ontology for DMRs with GREAT. The top 5 terms with corresponding binomial FDR values were shown.

Supplemental Figure 5

A. IRF8cKO deletion efficiency. Quantitative PCR data to estimate deletion efficiency in IRF8cKO (n=17 for P12; n=5 for 4wk). Using standard curve methodology, we quantified a copy number of the region to be deleted (IRF8 exon2) in sorted microglia. The efficiency was calculated as (IRF8 exon2 copies/control region copies), where 1.0 stands for no deletion.
B. Overlap of DEGs in IRF8KO and IRF8cKO microglia.

Supplemental Figure 6

C. UMAP depicting a population expressing Ly6c2 mRNA
D. Overlap of DEGs between bulk RNA-Seq and scRNA-Seq used in this paper.
E. GO analysis of 548 genes enriched in IRF8KO Cluster 4 compared to the other IRF8KO clusters. The top 5 categories are shown. See Supplemental Methods for identifying DEGs from scRNA-seq data.

Supplemental Figure 7

A. Volcano plot for RNA-Seq comparing WT and 5xFAD/WT microglia. Genes upregulated in 5xFAD/WT microglia over WT are set as a 5xFAD associated gene set, colored in pink (n=699, FDR<0.08). or genes downregulated in WT are colored in blue (n=137). Representative genes are labeled.
B. Heatmap showing expression of 5xFAD associated gene set in WT and IRF8cKO. Representative genes are labeled on the right.
C. Heatmap showing expression of 5xFAD associated gene set in WT and IRF8KO. Representative genes are labeled on the right.
D. Representative histology images depicting immunostaining of the cortex regions of 5xFAD/WT or 5xFAD/IRF8KO brain with 6E10 for Aβ and Iba1 (Axl) for microglia identification. Merge images on the right illustrate a lack of extensive colocalization in the 5xFAD/IRF8KO brain.
E. Gene expression levels of Trem2, Tyrobp, Tyro3, Axl, and Mertk genes in 3 months old WT and IRF8KO microglia. The log2 count+1 data from RNA-Seq were shown.

Supplemental Methods

Identification of non-super enhancer IRF8 cluster regions (IRF8nSEC)

To find the genomic regions occupied by dense, multiple IRF8 peaks similar to H3K27^{high} regions, we analyzed the intervals of IRF8 peaks within H3K27^{high} regions as shown in Figure S3C. Next, by taking advantage of the 75% Quantile value in Figure S3C, the peaks within 40kb intervals were grouped so that we could specify the regions occupied by IRF8 of similar linear density. To avoid the contamination of promoter IRF8, we removed the IRF8 peaks without H3K4me1 marks and the ones within 2.5kb from TSS in advance. ROSE was employed for grouping IRF8 peaks with *-s 40,000* option instead of *-s 12,500*. The grouped IRF8 cluster regions, except super enhancer regions, were defined as “non-super enhancer IRF8 cluster regions” (IRF8nSEC). The density of IRF8 peaks and region size were compared to the super enhancer regions by the Kolmogorov-Smirnov test (Figure S3C, S3D).

Identification of differentially expressed genes in scRNA-Seq clusters

The differential expression analysis for each cluster was performed by SigEMD[58], where the number of permutations was set to 100, and genes were filtered with p.adjust<0.01. The consistency between this DEG estimation and bulk RNA-seq transcriptome was examined in Supplemental Figure 5B. The genes enriched in IRF8 Cluster 4 were identified in comparison with the other IRF8 clusters.

Quantitative PCR to analyze the deletion efficiency by Cre recombinase

The genomic DNA from five to twenty thousand EYFP positive microglia was isolated with Quick-DNA Microprep plus kit (Zymo Research) as the manufacturer’s protocol. The DNA for the standard curve was prepared similarly from mouse cells. Quantitative PCR was performed using Fast SYBR Green Master Mix (Applied Biosystems) with the following primer set.

Control chr8 region Forward: 5'-TTTCAAGCCTTCTGTCTGCG-3'

Control chr8 region Reverse: 5'-CACCGCGTGCTCAACTACTC-3'

Irf8 exon 2 Forward: 5'-ATCGAACAGATCGACAGCAGC-3'

Irf8 exon 2 Reverse: 5'-CTTCCAGGGGATACGGAACA-3'

Supplemental Reference

1. Wang, T. & Nabavi, S. SigEMD: A powerful method for differential gene expression analysis in single-cell RNA sequencing data. *Methods* 145, 25-32 (2018).

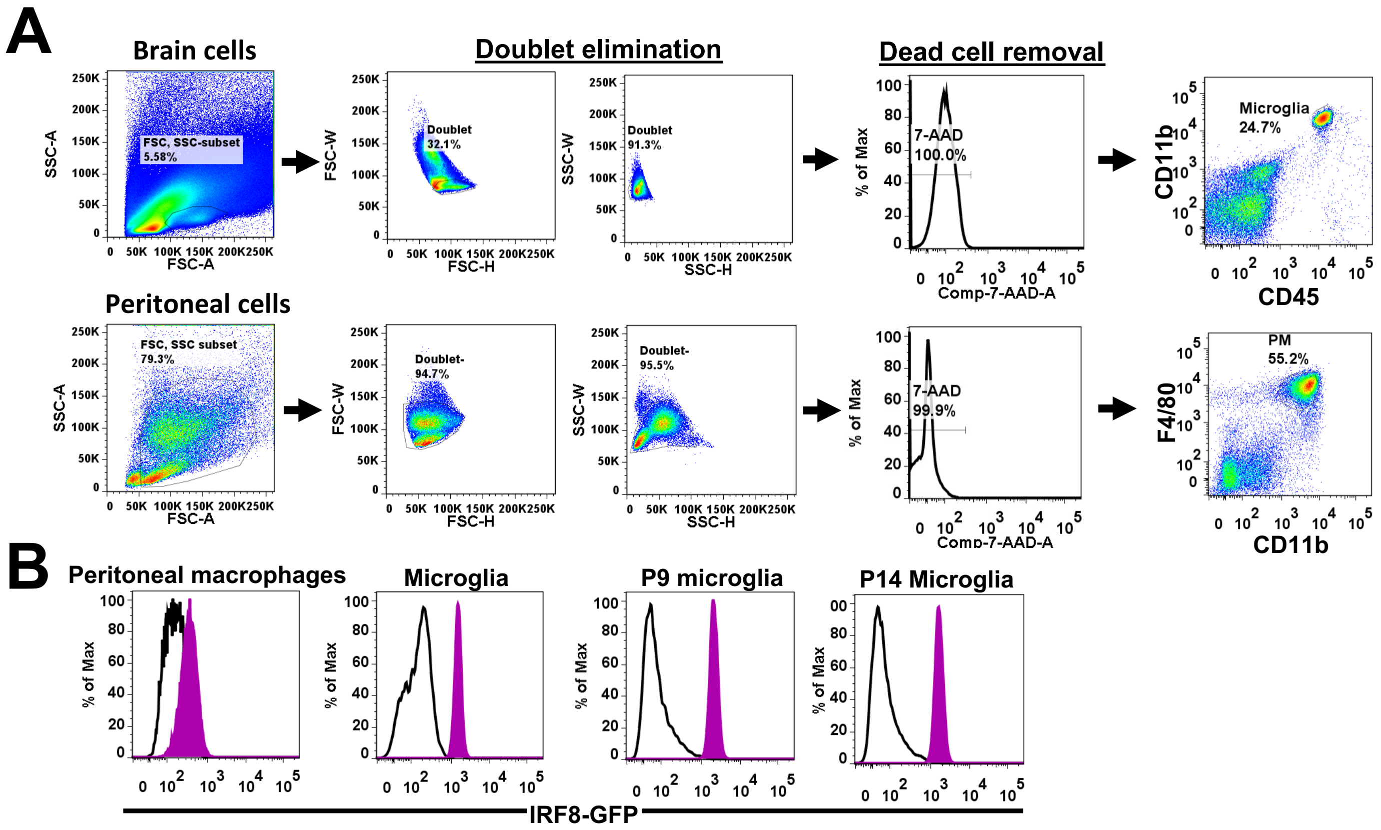

Fig.S1 Saeki et al.

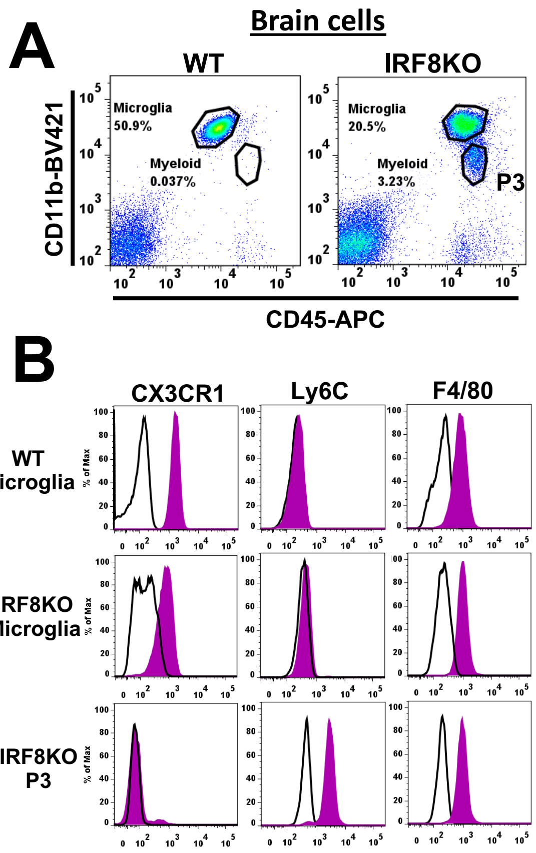

Fig.S2 Saeki et al.

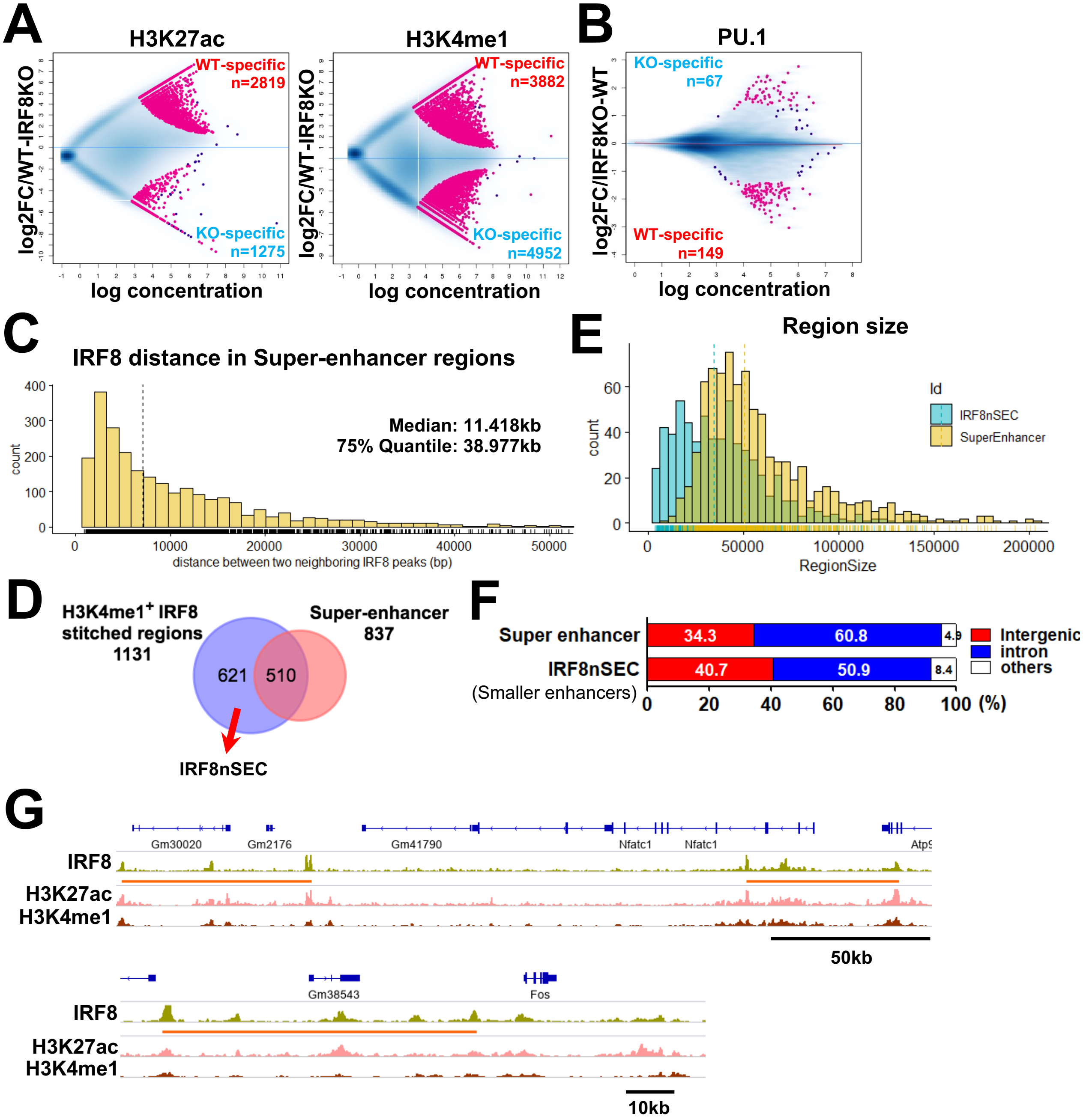

Fig.S3 Saeki et al.

A

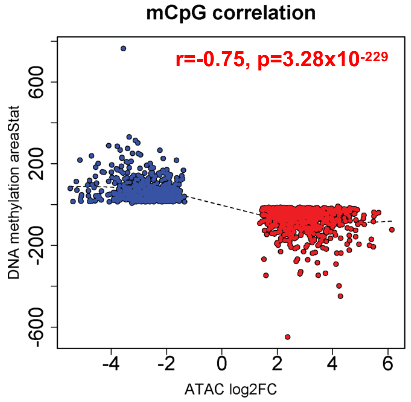

B

Demethylated in WT microglia

#Term Name	FDR
immune response	1.26E-15
leukocyte differentiation	2.49E-13
regulation of cell activation	2.66E-13
regulation of leukocyte activation	7.54E-13
adaptive immune response	7.46E-13

Methylated in WT microglia

#Term Name	FDR
negative regulation of smoothened signaling pathway	6.66E-17
positive regulation of Wnt signaling pathway	1.26E-11
regulation of Wnt signaling pathway	2.47E-11
regulation of smoothened signaling pathway	4.76E-11
embryonic digestive tract development	7.05E-11

Fig.S4 Saeki et al.

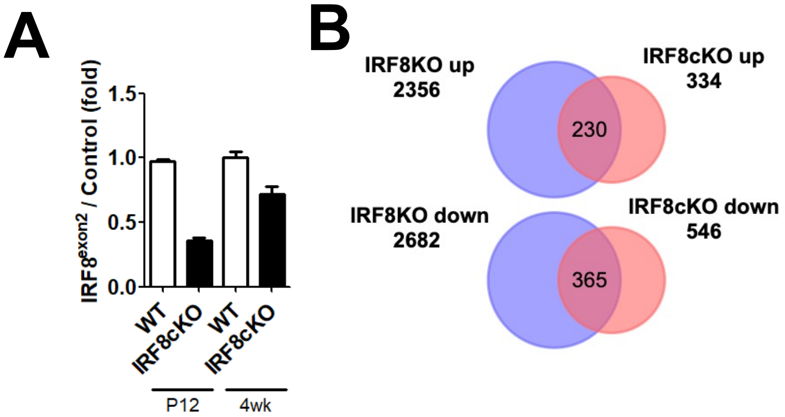

Fig.S5 Saeki et al.

A

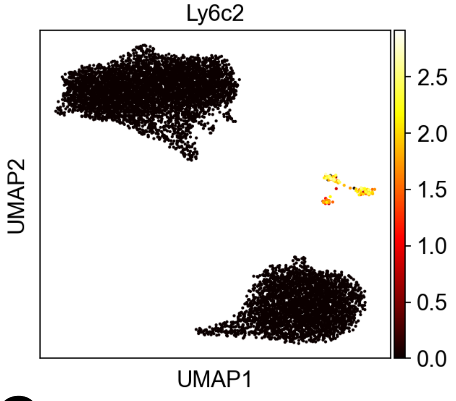

B

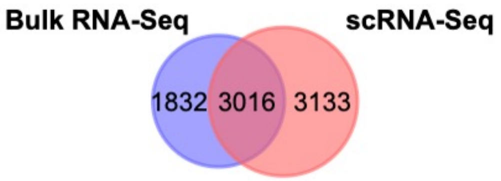

C

IRF8_CL4 vs rest of IRF8KO microglia

Term	Description	FDR
GO:0009615	response to virus	-50.564
GO:0035456	response to interferon-beta	-35.296
mmu05164	Influenza A - Mus musculus (house mouse)	-12.793
GO:0034341	response to type II interferon	-11.792
GO:0060759	regulation of response to cytokine stimulus	-11.513

Fig.S6 Saeki et al.

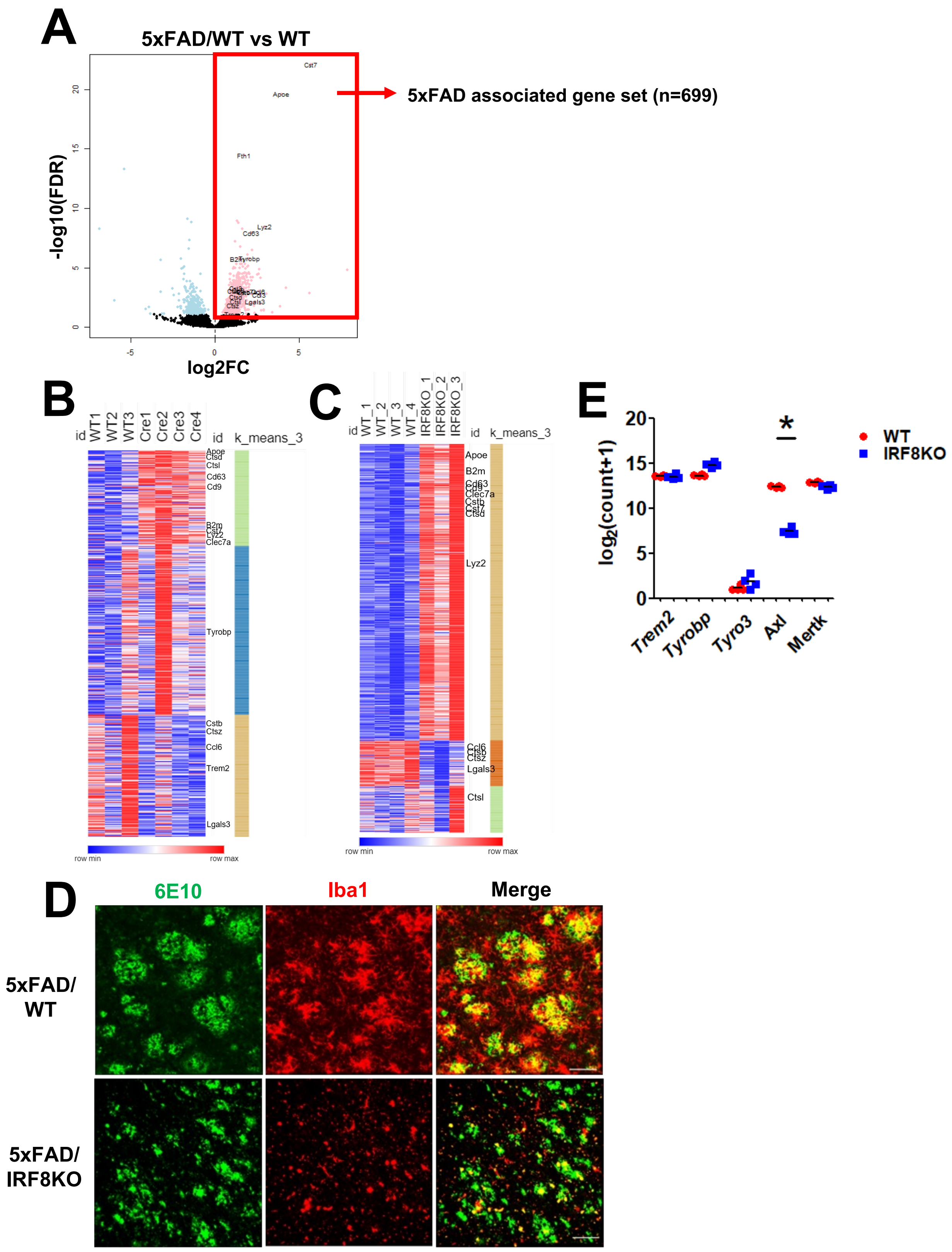

Fig.S7 Saeki et al.
